# Research Progress in Surface-Enhanced Infrared Absorption Spectroscopy: From Performance Optimization, Sensing Applications, to System Integration

**DOI:** 10.3390/nano13162377

**Published:** 2023-08-19

**Authors:** Dongxiao Li, Cheng Xu, Junsheng Xie, Chengkuo Lee

**Affiliations:** 1 Department of Electrical and Computer Engineering, National University of Singapore, Singapore 117583, Singapore; dongxiao@nus.edu.sg (D.L.); xucheng@u.nus.edu (C.X.); junshengxie@u.nus.edu (J.X.); 2 Center for Intelligent Sensors and MEMS (CISM), National University of Singapore, Singapore 117608, Singapore; 3 NUS Suzhou Research Institute (NUSRI), Suzhou 215123, China

**Keywords:** metamaterials, nanophotonics, surface-enhanced infrared absorption, plasmonic, light–matter interaction, sensor, chiral, machine learning, 2D material

## Abstract

Infrared absorption spectroscopy is an effective tool for the detection and identification of molecules. However, its application is limited by the low infrared absorption cross-section of the molecule, resulting in low sensitivity and a poor signal-to-noise ratio. Surface-Enhanced Infrared Absorption (SEIRA) spectroscopy is a breakthrough technique that exploits the field-enhancing properties of periodic nanostructures to amplify the vibrational signals of trace molecules. The fascinating properties of SEIRA technology have aroused great interest, driving diverse sensing applications. In this review, we first discuss three ways for SEIRA performance optimization, including material selection, sensitivity enhancement, and bandwidth improvement. Subsequently, we discuss the potential applications of SEIRA technology in fields such as biomedicine and environmental monitoring. In recent years, we have ushered in a new era characterized by the Internet of Things, sensor networks, and wearable devices. These new demands spurred the pursuit of miniaturized and consolidated infrared spectroscopy systems and chips. In addition, the rise of machine learning has injected new vitality into SEIRA, bringing smart device design and data analysis to the foreground. The final section of this review explores the anticipated trajectory that SEIRA technology might take, highlighting future trends and possibilities.

## 1. Introduction

Infrared spectroscopy is a powerful tool for material characterization, allowing non-destructive, label-free access to information about the chemical structure and species of a molecule. Based on these inherent properties, infrared spectroscopy has unlocked numerous breakthrough applications in fields such as biomedicine and environmental monitoring. However, the small infrared absorption cross-section of molecules limits the application of traditional infrared spectroscopy in the field of trace molecular detection. This limitation can be explained by the Beer–Lambert law (also known as Beer’s law) [1]. The general expression of the Beer–Lambert law is:

(1) A=εcl

where *A* is the absorbance, *ε* is the molar absorptivity (also known as the molar absorptivity), *c* is the concentration of the substance, and *l* is the path length of light traveling through the substance. According to Equation (1), it can be seen that the absorbance *A* is proportional to the thickness (*l*) of the absorbing material and the concentration (*c*) of the absorbing substance. When the thickness of the analyte is very thin or the concentration is very low, traditional spectrometers cannot obtain observable absorbance. In response to this problem, a new strategy is urgently needed to enhance the detection sensitivity of traditional infrared spectrometers and promote the development of infrared spectroscopy technology.

There are several methods to overcome the limitations mentioned above, such as optimizing the infrared light source, developing ultra-sensitive infrared detectors, and utilizing the Surface-Enhanced Infrared Absorption (SEIRA) effect [2]. While brighter infrared light sources and more sensitive infrared detectors can enhance the detection sensitivity of infrared spectrometers, they often come at higher costs. In contrast, SEIRA spectroscopy is a low-cost and effective method to enhance the interaction between light and matter [3]. In 1980, Hartstein et al. first proposed the concept of SEIRA [4]. By using randomly arranged silver nanoparticles, they demonstrated a 20-fold enhancement in the infrared vibrational signal of a monolayer molecular film. They proposed that the enhancement originated from “collective electronic resonances excited by the incident light”. Today, the underlying mechanism of this enhancement is well understood and is associated with Surface Plasmon Polaritons (SPP) [5], which are typically combined with Attenuated Total Reflection (ATR) techniques [6].

However, the development of SEIRA has been relatively slow in the nearly 30 years since 1980. There are likely two factors contributing to the slow progress of SEIRA. On the one hand, the SEIRA enhancement based on metal island films is a non-resonant enhancement mechanism. It does not tune the plasmon resonance to the infrared range, resulting in small enhancement factors typically ranging from 10^1^ to 10^2^ [4,7,8]. This is less impressive compared to Surface-Enhanced Raman Spectroscopy (SERS), which can achieve enhancement factors as high as 10^3^ during the same period [9,10]. On the other hand, metal island films, which are prepared by gas-phase or electrochemical deposition methods due to the limitations of micro/nanofabrication techniques, exhibit strong structural variations. This randomness in the structural properties of the metal island films makes the SEIRA enhancement signal highly unstable.

The emergence of metamaterials has brought new opportunities for the sensitivity and stability of SEIRA spectroscopy [11,12,13,14,15]. Metamaterials are a class of artificially designed materials with subwavelength periodic structures that allow for arbitrary manipulation of incident electromagnetic waves [16]. They exhibit extraordinary physical properties that are not found in natural materials, such as negative refractive index [17], near-field enhancement [18], Electromagnetically Induced Transparency (EIT) [19], Electromagnetically Induced Absorption (EIA) [20,21], inverse Doppler shift effect [22,23], and inverse Cherenkov effect [24]. Based on these extraordinary physical characteristics, a wide range of new application areas have emerged, including superlenses [25,26], slow light [27], nonlinear optics [28], holography imaging [29], invisibility cloaking [30], and sensing [31]. In particular, research on sensing based on metamaterials has become a major focus in recent years [32,33,34].

By customizing the size of metamaterial nanoantennas, the resonant frequency can be set in the mid-infrared range while generating strong and highly confined electromagnetic field hotspots. These intense electromagnetic field hotspots enable strong interactions with neighboring analytes, significantly enhancing the sensitivity of SEIRA spectroscopy [35]. Therefore, metamaterials have become an ideal choice for biochemical sensing and spectroscopic applications [36]. Furthermore, the advancement of customizable metamaterials and micro/nanofabrication techniques has improved the randomness in the variations of metal island films, laying the foundation for achieving stable SEIRA spectroscopy enhancement.

In 2008, Neubrech et al. first demonstrated the significant SEIRA spectroscopy enhancement effect of plasmonic nanoantennas [37]. In this work, Neubrech et al. achieved Localized Surface Plasmon Resonance (LSPR) in the infrared region by artificially designing the dimensions of the plasmonic nanoantennas. A monolayer of octadecanethiol (ODT) molecules was used as the target analyte. Significant SERIA enhancement was observed when the resonance frequency of the analyte matched the plasmonic resonance frequency. However, the enhancement effect of individual nanoantennas was limited. In order to further improve SEIRA performance, Adato et al. proposed the use of arrayed plasmonic nanoantennas for ultra-sensitive spectral detection of protein monolayers [38]. Compared to individual nanoantennas, the arrayed nanoantennas can excite collective electron resonances, resulting in larger local field enhancements and sharper spectral responses. Experimental results demonstrated that the absorption signal from the arrayed nanoantennas far exceeded that of individual nanoantennas, potentially achieving zeptomole-level protein detection limits. Furthermore, compared to individual nanoantennas and chemically prepared metal island films, the arrayed nanoantennas exhibited higher reliability and repeatability. Since then, SEIRA technology has experienced rapid development.

**Figure 1:**
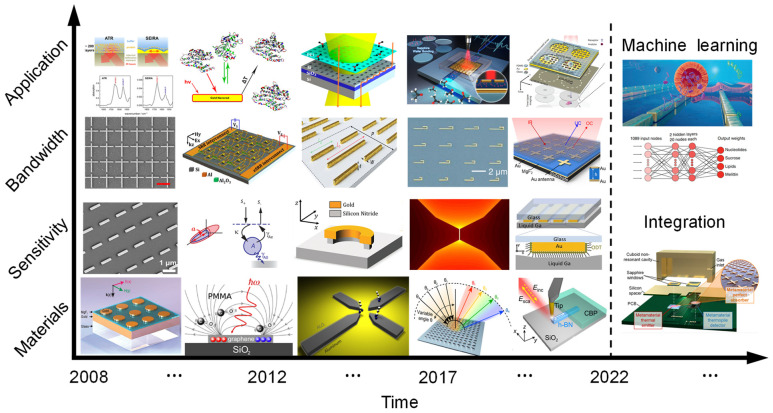
The roadmap of SEIRA based on IR metamaterials over the last 15 years. Reprinted from ref. [39] with permission, Copyright@2010 American Chemical Society; Reprinted from ref. [38] with permission, Copyright@2009 National Academy of Science; Reprinted from ref. [40] with permission, Copyright@2012 American Chemical Society; Reprinted from ref. [41] with permission, Copyright@2008 National Academy of Science; Reprinted from ref. [42] with permission, Copyright@2014 American Chemical Society; Reprinted from ref. [43] with permission, Copyright@2013 American Chemical Society; Reprinted from ref. [44] with permission, Copyright@2016 American Chemical Society; Reprinted from ref. [45] with permission, Copyright@2014 Wiley-VCH; Reprinted from ref. [46] with permission, Copyright@2015 Optical Society of America; Reprinted from ref. [47] with permission, Copyright@2018 Springer Nature; Reprinted from ref. [48] with permission, Copyright@2014 American Chemical Society; Reprinted from ref. [49] with permission, Copyright@2017 American Chemical Society; Reprinted from ref. [50] with permission, Copyright@2018 American Chemical Society; Reprinted from ref. [51] with permission, Copyright@2019 AAAS; Reprinted from ref. [52] with permission, Copyright@2012 Springer Nature; Reprinted from ref. [53] with permission, Copyright@2020 American Chemical Society; Reprinted from ref. [54] with permission, Copyright@2023 Wiley-VCH; Reprinted from ref. [55] with permission, Copyright@2022 Wiley-VCH; Reprinted from ref. [56] with permission, Copyright@2022 Springer Nature; Reprinted from ref. [57] with permission, Copyright@2020 American Chemical Society; Reprinted from ref. [58] with permission, Copyright@2023 Springer Nature. The scale bar is 2 μm.

This review summarizes the development of SEIRA technology and identifies four main trends: materials, sensitivity enhancement, enhanced bandwidth, and applications (Figure 1). In recent years, the Internet of Things (IoT), sensor networks, and wearable devices have presented new demands for the miniaturization and system integration of infrared spectroscopy systems and chips. Therefore, achieving miniaturization and system integration of infrared spectroscopy has become a crucial path for the development of SEIRA technology. Additionally, the rise of Machine Learning (ML) has injected new vitality into SEIRA technology. Leveraging ML can enable more intelligent device design and data analysis. The final section of this review discusses perspectives on future trends in SEIRA technology development.

## 2. Resonator Materials

### 2.1. Metal Materials

**Figure 2:**
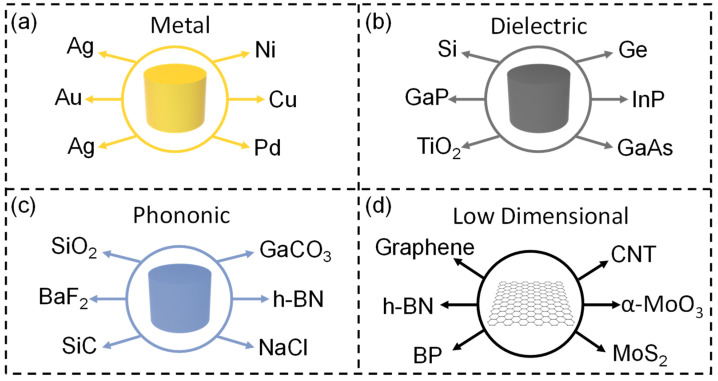
Choice of materials for SEIRA. (**a**) Metal materials. (**b**) Dielectric materials. (**c**) Phonon resonance materials. (**d**) Low-dimensional van der Waals materials.

Metal materials are among the most commonly used materials in SEIRA (Figure 2a). Metal surfaces possess high conductivity and exhibit Surface Plasmon Resonance (SPR) effects, making them exhibit excellent optical properties in the infrared range. This effect leads to highly concentrated electric fields on the metal surface at the nanoscale, enhancing the interaction between electromagnetic waves and molecules attached to the metal surface. In addition, gold’s chemical inertness and easy surface functionalization [59,60] also make it a versatile material suitable for biosensing [45,61,62,63]. Gold-based metamaterials have been demonstrated to be applicable to other wavelength ranges as well, including visible light [64], terahertz [65], and microwaves [66]. However, gold, as a precious metal, faces sustainability and cost challenges when producing SEIRA chips on a large scale.

In addition to gold, other metal materials such as silver [67,68,69,70], copper [71,72], titanium [73,74], palladium [75,76,77,78], and aluminum [44,79,80,81,82] can also excite plasmonic resonances. Aluminum, in particular, has recently attracted significant attention as an alternative to precious metal materials. Aluminum possesses several attractive features, including low cost, abundant reserves, compatibility with Complementary Metal-Oxide-Semiconductor (CMOS) processes, and support for resonances across an ultra-wide spectral range from ultraviolet to infrared [79,83]. Furthermore, aluminum spontaneously forms a native oxide layer of 2–4 nm thickness in atmospheric conditions. Compared to Au or Ag, the native oxide layer enables a wider range of covalent bonding schemes between molecules and antennas [44,84]. Therefore, aluminum is a highly regarded potential candidate material for SEIRA.

### 2.2. Dielectric Materials

Metal materials have played a key role in advancing the field of SEIRA. However, the inherent Ohmic losses of metals also limit their resonance linewidth, resulting in low-quality (Q) factors of the resonances [85]. Additionally, the high absorbance of metals may cause undesirable local heating, leading to analyte denaturation and hindering the development of in vivo sensing [48,86,87]. To overcome these limitations, dielectric materials with a high refractive index and low loss have emerged as an alternative to metals [88,89,90,91,92]. Numerous studies have shown that dielectric resonators can support various electric and magnetic Mie-type resonance modes that occur at different wavelengths in the scattered light spectra, allowing for precise spectral control of the system’s electric/magnetic response [93,94,95,96].

Recently, nanosystems based on dielectric materials have provided a platform for achieving high-Q factors and ultra-sharp resonances [97,98,99]. These ultra-sharp resonances offer new possibilities for highly sensitive nanophotonic sensing [100,101,102]. For example, Ghofraniha et al. realized a high-Q microlaser for low-concentration biosensing using the free-space whispering gallery mode [103]. Furthermore, designing high-Q resonances narrower than molecular vibrational bands enables monochromatic SEIRA sensing of specific target analytes without the need for an infrared spectrometer [51,85,102]. Common dielectric materials currently used include Silicon (Si) [102], Germanium (Ge) [51,85], Gallium Phosphide (GaP) [104], Indium Phosphide (InP) [105], and others (Figure 2b). These materials possess high refractive indices, low losses, and excellent optical properties, making them widely applicable choices in the fields of spectroscopy and nanophotonics. Additionally, doping provides a new dimension for dielectric resonator design. An exciting benefit of doping is the possibility of electronic tuning of resonances [106], which differs from metals that require resizing the resonators for each target spectral position. As materials science and nanotechnology continue to advance, research on dielectric materials will continue to deepen, bringing forth more innovations and opportunities for applications in biomedicine and optoelectronics.

### 2.3. Phonon Resonance Materials

In recent years, phonon resonance materials have attracted widespread attention. Unlike LSPR in metallic materials, phonon resonance materials can couple light to lattice vibrations known as phonons. Specifically, in ionic solids composed of positively and negatively charged “ions”, the heterogeneous motion of “atoms” in the lattice can strongly couple with the electromagnetic field. The coupling between light and lattice vibrations gives rise to Phonon-Polariton (PhP), which is a quasi-particle resulting from the coupling of photons and optical phonons. PhPs have been observed in the Reststrahlen band of polar crystals, spanning from Transverse Optical (TO) to Longitudinal Optical (LO) phonon frequencies [107]. Due to the inherent low damping rates and strong light confinement associated with phonon scattering, PhP modes exhibit higher Q factors and Purcell enhancement compared to plasmon polaritons [108,109,110,111]. These advantages provide new opportunities to explore the coupling dynamics between PhP modes and other collective oscillations [112,113].

For instance, Hu et al. investigated the strong coupling between propagating and localized PhP modes supported by Silicon Carbide (SiC) nanorod lattices using near-field nanoscale FTIR spectroscopy [112]. They revealed the evolution of mode hybridization with lattice constants by acquiring near-field spectra at local points on the nanorod lattice and observing pronounced energy-splitting gaps in the Rabi splitting spectra.

The strong coupling between light and phonons also offers interesting possibilities for high-performance sensing platforms. Liu et al. demonstrated the strong coupling between Surface Phonon-Polaritons (SPhPs) and molecular vibrations observed in far-field measurements using a single quartz microcylinder as an SPhP resonator [114]. Benefiting from high-Q factors and ultra-small mode volumes, SPhPs exhibited distinct mode splitting and anticrossing features with 4-nitrobenzyl alcohol molecules. This finding paves the way for enhancing vibrational strong coupling sensitivity and miniaturizing mid-infrared spectroscopy. This strong vibrational coupling has also been observed at other infrared frequencies in polar crystal materials such as SiO_2_ [115,116,117], SiC [110,112,118], Calcium Carbonate (CaCO_3_) [119], and Hexagonal Boron Nitride (hBN) (Figure 2c) [108,111].

### 2.4. Low-Dimensional van der Waals Materials

In addition to metals, dielectric materials, and phonon resonance materials, the extreme field confinement of low-dimensional van der Waals (vdW) materials also brings exciting prospects for nanophotonics and infrared sensing [120]. In infrared sensing, the two most extensively studied low-dimensional vdW structures are one-dimensional (1D) carbon nanotubes [121,122] and two-dimensional (2D) graphene (Figure 2d) [42,123]. For example, single-walled carbon nanotubes have been used for single-molecule detection [124] and in vivo detection [125] through exciton effects. Two-dimensional vdW materials, such as graphene, have been shown to enhance plasmonic field confinement more effectively than metal nanostructures. Additionally, graphene plasmons demonstrate unique potential in dynamic tunable infrared absorption spectroscopy for detecting molecular structural changes and vibrational mode fingerprinting [126,127,128]. Subsequently, Hu et al. demonstrated in situ electrical tuning of graphene plasmons across the fingerprint region using mid-infrared resonant graphene nanoribbons [129]. The highly confined graphene plasmon polaritons achieved an extremely high detection sensitivity at the sub-monolayer level. By utilizing the strong near-field component perpendicular to the graphene direction, the authors detected out-of-plane and in-plane vibrational modes that are inaccessible with conventional Fourier Transform Infrared (FTIR) measurements. They also achieved label-free identification of gas molecules adsorbed on the graphene surface using graphene nanoribbons, detecting concentrations as low as 800 zmol/μm^2^ [130].

Although the extreme optical confinement of 2D materials is an attractive feature for sensing, a drawback is the weak coupling efficiency between external light and graphene plasmon polaritons [131]. This results in typically low extinction values (below 5%), which is impractical for device applications [120]. Recent efforts have been made to enhance the plasmonic response by utilizing multilayer stacking [132], integration with photonic cavities (i.e., Fabry–Perot) [133,134], and hybrid substrates containing plasmonic nanostructures [135,136]. For instance, Nong et al. explored graphene plasmons in multilayer graphene nanoribbons [134]. By incorporating Fabry–Perot-type cavities, they achieved significant improvements in localized graphene plasmon absorption, increasing it from 3% to over 92%. The performance of the improved SEIRA is an order of magnitude higher than that of single-layer graphene nanostructures.

### 2.5. Hybrid Materials

Although the aforementioned materials can be used to fabricate metamaterials, each material has its own limitations. By combining two or more of these materials to form new structures, the shortcomings of each material can be overcome, and new properties can emerge [137]. These properties offer new opportunities for controlling light propagation and infrared sensing. For example, Lee et al. demonstrated a graphene acoustic plasmonic resonator by integrating graphene with ultra-flat metal strips to overcome the momentum mismatch barrier between acoustic plasmons in graphene and the excitation source [136]. It exhibits near-perfect absorption (94%) of incident mid-infrared light and enhances the light–matter interaction. This graphene acoustic plasmonics can sensitively measure absorption bands of proteins at angstrom thickness and surface phonon modes in SiO_2_.

In addition, hybrid metal-dielectric nanostructures have recently received attention [138]. For instance, Ray et al. designed a hybrid metal-dielectric nanoscale antenna [139]. The hybrid nanoscale antenna consists of an aluminum disk and a silicon cylinder separated by a SiO_2_ spacer. This metal-dielectric hybrid design combines the strong field enhancement of plasmonic metals with several low-loss radiative channels of dielectric resonators. The coupling between different materials endows it with a combination of desirable qualities and superior optical response. Through further optimization, the hybrid metal-dielectric nanostructure achieves a refractive index sensitivity of 245 nm/RIU for bulk refractive index sensing.

Furthermore, combining metal nanoparticles with semiconductor nanoparticles can also generate quantum mechanical effects. For example, Huang et al. designed a plasmonic nanocavity based on the coupling between gold and CdO nanocrystals [140]. The subnanometer gap between the gold and CdO nanocrystals creates a quantum mechanical tunneling effect. The quantum mechanical tunneling effect leads to a resonant blue shift of the Au-CdO nanocrystals and promotes field enhancement and increased SEIRA signals.

## 3. SEIRA Sensitivity

### 3.1. Structural Optimization

Sensitivity is one of the key parameters used to evaluate the performance of SEIRA. Typically, the Enhancement Factor (EF) is used to evaluate the sensitivity of SEIRA [141]. *EF* relates the enhanced signal intensity to the signal obtained using standard infrared techniques (transmission, reflection), and its expression is as follows:

(2) $$EF=\frac{I_{SEIRA}/I_{0}}{A_{SEIRA}/A_{0}}$$

where *I_SEIRA_* is the enhanced signal intensity, and *I*_0_ is the signal intensity without enhancement. Additionally, *A_SEIRA_* and *A*_0_ represent the areas (volume) covered (filled) by the molecule in the SEIRA or reference measurement, respectively [142,143]. Since the enhanced SEIRA signal mainly originates from the molecules located in the hotspots of the antenna, *A_SEIRA_* is often approximated as the volume of the surface region at the tip of the antenna [144].

**Figure 3:**
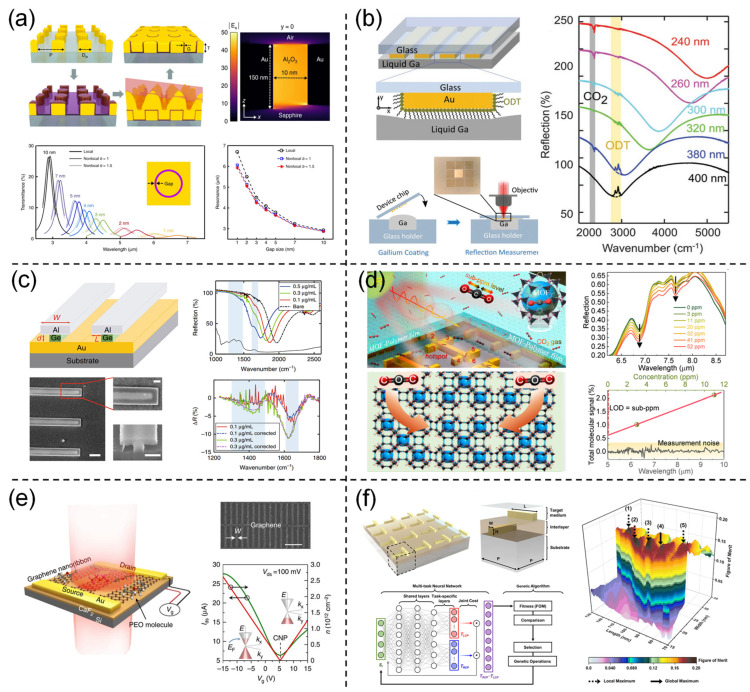
Structural optimization is used to enhance the sensitivity of plasmonic devices. (**a**) Extreme-scale nanophotonic devices with critical gap dimensions of just 1–2 nm Reprinted from ref. [147] with permission, Copyright@2019 Springer Nature; (**b**) Structure of liquid-gallium-based SEIRA sensors. Reprinted from ref. [55] with permission, Copyright@2022 Wiley-VCH; (**c**) High-sensitivity nanophotonic sensors with passive trapping of analyte molecules in hot spots. Reprinted from ref. [152] with permission, Copyright@2021 Spring Nature, The scale bar in the left image is 2 μm, and the scale bars in the right two images are 600 nm; (**d**) MOF/polymer hybrid thin films for gas molecule enrichment. Reprinted from ref. [157] with permission, Copyright@2022 Springer Nature; (**e**) Graphene plasmon enhanced molecular fingerprint sensor. Reprinted from ref. [129] with permission, Copyright@2016 Springer Nature; (**f**) Structure of the forward-prediction network. Reprinted from ref. [160] with permission, Copyright@2023 American Chemical Society.

The sensitivity and detection limit of SEIRA strongly relies on the near-field intensity of the metamaterial [145]. Currently, a common method used to enhance near-field intensity is by reducing the gap between adjacent nanoscale antennas. The small gap increases the coupling between neighboring antennas and generates higher field enhancement within the nanoscale gap. For example, Dong et al. employed plasmonic junction nanoscale antennas with gaps smaller than 3 nm, aiming to achieve a theoretical SEIRA EF of 10^7^ [49]. Optimized nanoscale junction antennas with ultra-small nanogaps enabled the detection of as few as 500 molecules of 4-nitrophenol. To achieve large-scale fabrication of nanogaps, Yoo et al. proposed a high-throughput, batch fabrication method based on atomic layer lithography to create a coaxial nanohole array [146]. This technique utilizes Atomic Layer Deposition (ALD) with angstrom-level thickness resolution to create narrow (as low as 1 nm) and long (up to several centimeters) slits (Figure 3a) [147]. The ultra-small nanogaps offer opportunities for strong light–matter coupling and ultra-sensitive molecular sensing [115]. Studies have shown that with a nanogap size of 7 nm, an EF of 5 × 10^5^ was achieved for detecting 5 nm silk protein [143].

In addition, creating vertical nanogaps can also be used to enhance near-field intensity [148]. Vertical nanogaps are typically employed in metamaterial absorbers where a metal film is present. The nanoscale vertical gaps confine the light strongly between the metal antennas and the metal film. However, the dielectric layer between the nanoscale antennas and the metal film hinders the chance of molecular overlap with the near field. One effective approach to address this is by using microchannels as a replacement for the dielectric layer [53,149]. For example, Le et al. proposed a plasmonic-nanofluidic metamaterial composed of plasmonic resonators and a metal film sandwiched between nanofluidic channels [150,151]. This structure enables controllable and efficient transport of molecules between the top resonator and the bottom metal mirror, thereby enhancing the infrared absorption signal of the molecules. However, as microchannels shrink to the nanoscale, it becomes increasingly challenging to transport analyte molecules into these gaps (i.e., hotspots). Especially when the gap size is comparable to the typical size of the molecules. This issue fundamentally limits further improvement in the performance of nanophotonic sensors. To facilitate the transfer of analytes to the gaps between the antennas and the metal film, Miao et al. developed a chip-based SEIRA sensor using liquid metal (Figure 3b) [55]. The sensor consists of an array of metal nanobands, which are separated from a nano-dielectric layer by a metal ground plane, essentially forming a nanoscale chip antenna array. The analyte is physically/chemically adsorbed onto the metal nanobands, serving as the nano-dielectric layer. Subsequently, liquid gallium is added to cover the analyte molecular film and serve as the ground plane for the nanoscale chip antenna. Due to the highly confined and enhanced electric field in the nanogap between the metal nanobands and the liquid gallium, the molecular vibrational signal associated with the analyte film can be significantly enhanced. Importantly, the liquid gallium can be easily removed from the sensor surface after measurement, making such sensors readily reusable.

Improving the spatial overlap between molecules and hotspots is also crucial for SEIRA sensitivity. However, the near-field enhancement of nanoscale antennas can partially enter the dielectric layer, hindering the probability that molecules and hotspots will overlap. Etching the dielectric layer to prepare nanopedestals is an effective way. For example, Cetin et al. prepared dielectric nanopedestals through isotropic fabrication techniques and fabricated polarization-insensitive mid-infrared nanoring antennas on nanopedestals [45]. The nanopedestals expose the hotspots of the top antenna to free space, thus providing maximum overlap between the target biomolecule and the plasmonic hotspots. The increased spatial overlap enhances SEIRA sensitivity, resulting in a sensitivity improvement of 2.5 to 10 times compared to nanoscale antennas on a substrate. Additionally, dielectric nanopedestals with nanogrooves can passively capture and concentrate analyte solutions. For instance, Miao et al. developed metal-insulator–metal optical resonant cavities with nanopedestals (Figure 3c) [152]. The width of the dielectric nanopedestals is smaller than that of the top metal nanoscale antennas by several hundred nanometers, creating nanogrooves on both sides of each dielectric nanopedestal. When an analyte solution is loaded onto the device surface, it covers the entire array of resonators and infiltrates the nanogrooves. Subsequently, the solvent gradually evaporates, causing the analyte to precipitate and deposit inside and near the grooves. The passive capture of molecules by the nanopedestals further enhances SEIRA sensitivity.

Although nanopedestals enable the passive capture of molecules, this functionality is ineffective for discrete gas molecules. The smaller intermolecular forces of gas molecules result in their random distribution in free space. However, the limited near-field enhancement of nanoscale antennas restricts their ability to sense more gas molecules [50,127,153,154,155]. This issue can be addressed by employing molecular enrichment membranes through physical or chemical adsorption. For example, Zhou et al. used ZIF-8 to trap CO_2_ and CH_4_ within molecular cages [156]. This strategy enhances the spatial overlap between molecules and hotspots, providing a unique opportunity for gas molecule detection. Subsequently, Zhou et al. achieved sub-parts-per-million (sub-PPM) detection limits for gas detection using chemical/physical synergistic adsorption (Figure 3d) [157]. Another approach similar to molecular enrichment membranes is target molecules, which can specifically adsorb proteins, nucleic acids, and lipids in a liquid-phase environment [47,61,62,158,159]. In the presence of target molecules, the captured biomolecules overlap with hotspots, enabling the detection of biomolecules at low concentrations.

In addition to structural optimization, there are new approaches that can enhance sensing sensitivity. For example, Tittl et al. proposed a design scheme for pixelated all-dielectric metasurfaces (Figure 3e) [85]. This scheme exploits the collective behavior of Mie resonances, which can be identified as supercavity modes driven by Bound States in Continuum (BIC) physics. In this mode, the metasurface exhibits high-Q characteristics, enabling attractive light–matter interactions. Research has shown that the design of all-dielectric high-Q metasurfaces exhibits strong vibrational enhancement, improving sensing performance by an order of magnitude compared to widely used metal antenna geometries. Additionally, Rodrigo et al. fabricated mid-infrared plasmonic biosensors using graphene nanoribbons [126]. The extreme spatial confinement of graphene enables unprecedented high overlap with nanoscale molecules, resulting in superior sensitivity in detecting their refractive indices and vibrational fingerprints (Figure 3e) [129]. The introduction of artificial intelligence algorithms and deep learning also brings new opportunities for enhancing SEIRA sensitivity [35,160,161,162,163,164,165]. Algorithm-driven self-iteration allows for the discovery of non-intuitive, irregularly shaped photonic structures that outperform empirically designed sensitivity in sensing applications [160,166]. For example, Han et al. developed an optimization solution combining deep learning and genetic optimization algorithms (Figure 3f) [160]. This solution utilizes deep learning for reverse design and optimization to achieve chiral plasmonic sensors with maximum sensitivity. Furthermore, ML can also perform rapid analysis and automate data processing, thereby enhancing SEIRA sensitivity [52,63,167].

While simple structural optimization can enhance SEIRA sensitivity, it falls short of detecting infrared vibrational information at nanoscale spatial resolution. Infrared scattering-type Scanning Near-Field Optical Microscopy (s-SNOM) was initially developed to realize nanoscale infrared spectroscopy [168]. In s-SNOM, a sharp metallic nanotip is brought near the sample and illuminated with incident infrared light. By measuring the amplitude and phase of scattered light generated from confined and enhanced near-field excitation at the apex of the metallic tip, information about the sample’s refractive index and absorption coefficient is obtained [169]. Although s-SNOM achieves near-field spectroscopy with nanoscale-sized probes, directed delivery of molecules into plasmonic hotspots remains a challenging task. This challenge is addressed by employing Atomic Force Microscopy-based Infrared Spectroscopy (AFM-IR) [170]. AFM-IR leverages the inherent near-field enhancement of SEIRA by the AFM tip itself, obviating the need for targeted molecular delivery and enabling flexible detection of molecules at any location on the substrate. Unlike s-SNOM, AFM-IR utilizes a pulse-wavelength-tunable infrared laser source to excite the infrared absorption of the sample. The sample absorbs the infrared pulsed beam, causing heating and thermal expansion. As the tip approaches the sample, the thermal expansion of the sample causes a mechanical vibration of the tip with an amplitude proportional to the local infrared absorption of the sample. Therefore, by measuring the tip amplitude while scanning the pulsed infrared laser, the infrared absorption spectrum of the sample can be obtained [171]. At present, tip-enhanced infrared spectroscopy exhibits extremely high sensitivity and is expected to realize single-molecule detection at the nanoscale [172,173].

### 3.2. Loss Optimization

Although structural optimization has greatly improved SEIRA’s performance, it comes at the cost of higher manufacturing expenses. This trade-off between performance and manufacturing costs hinders further optimization and widespread application of SEIRA devices [174]. In addition to the methods mentioned above, adopting loss engineering to enhance SEIRA performance is a promising solution that has gained significant attention in recent years. The underlying physical framework of loss engineering is Coupled Mode Theory (CMT). CMT was first proposed by Pierce [175] and Miller [176] at Bell Laboratories in the early 1950s to study the coupling behavior between two or more electromagnetic wave modes. Subsequently, Schelkunoff rigorously derived the equations of CMT using mode expansions, laying the theoretical foundation for CMT’s development [177]. In 1984, Hermann A. Haus extended CMT to the coupling between two resonators and determined that the solutions of the coupled system are time-dependent, leading to the development of Temporal Coupled-Mode Theory (TCMT) [178]. From the late 20th century to the early 21st century, the resurgence of nanophotonics has rekindled researchers’ interest in TCMT. In 2003, Fan et al. successfully used TCMT to explain Fano resonances in photonic crystal slabs [179]. Currently, TCMT has become a powerful physical model for analyzing electromagnetic wave propagation and describing light–matter interactions. It is widely employed to understand the coupling between waveguides and resonators and the various physical effects resulting from the coupling.

**Figure 4:**
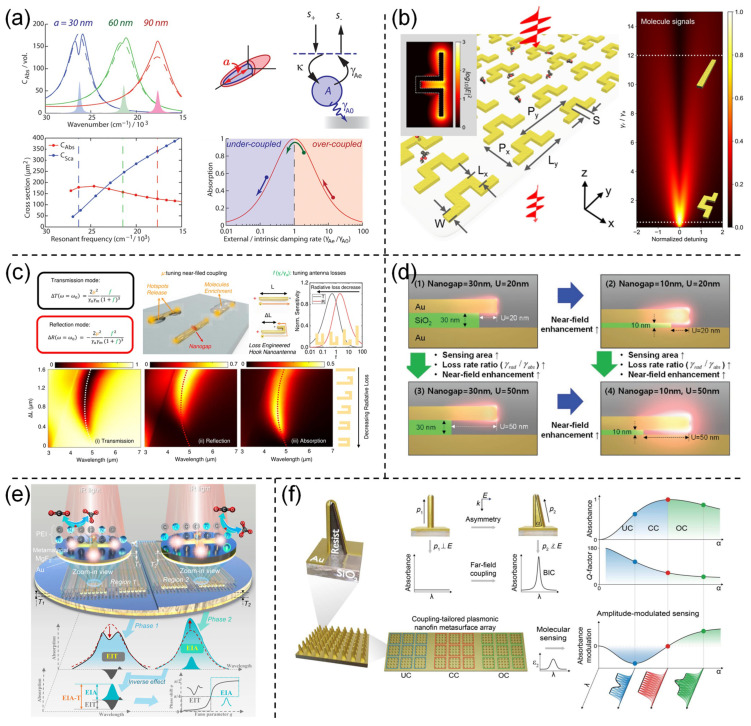
Loss optimization is used to enhance the sensitivity of plasmonic devices. (**a**) Engineered absorption enhancement and induced transparency in coupled molecular and plasmonic resonator systems. Reprinted from ref. [43] with permission, Copyright@2013 American Chemical Society; (**b**) Loss engineering optimization for ultrasensitive transmission infrared spectroscopy. Reprinted from ref. [174] with permission, Copyright@2019 American Chemical Society; (**c**) Loss engineering optimization for ultrasensitive reflectance infrared spectroscopy. Reprinted from ref. [52] with permission, Copyright@2022 Springer Nature; (**d**) Loss engineering combined with vertical nanogap for ultrasensitive molecular detection. Reprinted from ref. [181] with permission, Copyright@2021 Wiley-VCH; (**e**) Loss-induced phase transition in mid-infrared plasmonic metamaterials for ultrasensitive vibrational spectroscopy. Reprinted from ref. [182] with permission, Copyright@2022 Wiley-VCH; (**f**) Loss regulation in plasmonic bound states. Reprinted from ref. [183] with permission, Copyright@2022 AAAS.

Adato et al. utilized TCMT to demonstrate not only EIT but also EIA in coupled molecule-plasmon resonator systems (Figure 4a) [43]. To gain a deeper understanding, they employed bright and dark modes to comprehend the interactions in the plasmon-molecule coupling system. Using TCMT, they divided the loss of the bright mode into radiative loss (scattering) and intrinsic loss (absorption). The ratio between these two loss mechanisms (radiative damping constant and intrinsic damping constant γ*_Ae_*/γ*_A_*_0_) was found to be the main factor affecting the plasmonic band shape and absorption resonance. When the ratio is less than one, the resonator operates in an undercoupled mode (UC), resulting in a downward signal dip after coupling with the molecule (EIT). Conversely, when the ratio is greater than one, the device operates in an overcoupled mode (OC), leading to an upward signal peak after coupling with the molecule (EIA). Interestingly, if the addition of molecular damping causes the ratio between the radiative damping and intrinsic damping to approach unity (referred to as Critically Coupled (CC)), the absorption band of the plasmon remains nearly unchanged, neither decreasing nor increasing. This scenario is crucial for so-called perfect absorbers. This work using TCMT established the relationship between EIT/EIA signal responses and damping rates, providing an initial explanation for the interaction mechanism in plasmon-molecule coupling systems and laying the theoretical foundation for further research on light–matter interactions.

TCMT theory not only explains the mechanisms of light–matter interactions but also guides device design. Based on TCMT theory, optimal parameter configurations for achieving the best SEIRA performance can be broadly predicted, leading to the design of highly efficient SEIRA devices. Therefore, loss optimization holds the potential to obtain devices with optimal performance in a straightforward manner, thereby reducing device manufacturing costs. For example, Newman et al. investigated the role of plasmonic absorption and scattering in the generated SEIRA signal of antenna-coupled absorbers [180]. The study revealed that the optimal SEIRA signal can be achieved through transmission (extinction) measurements when the peaks of the absorption and scattering spectra of the antenna have similar amplitudes. Importantly, under the optimal conditions for SEIRA, the vibrational fingerprint is solely a result of scattering, with no contribution from absorption. This finding aligns with the discoveries made by Adato et al. [43] This work provides guidelines for controlling the scattering and absorption characteristics of plasmonics and demonstrates a preliminary approach for enhancing SEIRA sensitivity through loss optimization.

Although the concept of loss engineering in metamaterials has been reported, the full potential of reverse-engineering plasma nanoantennas based on loss engineering has not yet been fully explored. Therefore, Wei et al. optimized plasma nanoantennas using loss engineering to achieve ultra-sensitive transmission plasmonic molecular sensors (Figure 4b) [174]. Firstly, Wei et al. established a comprehensive theoretical framework for the coupled system using coupled mode theory. Secondly, guided by theoretical analysis, they predicted that reducing radiation losses would enhance the sensitivity of SEIRA devices. Within the theoretical framework, they designed crooked nanoantennas and straight nanoantennas. The crooked nanoantennas exhibited lower radiation losses compared to the straight nanoantennas. Experimental results demonstrated an EF of 2.8×10^4^ for the crooked nanoantennas, which is approximately 25 times higher than that of commonly used straight nanoantennas. This work provides a new dimension for the design of plasmonic molecular sensors. Subsequently, Ren et al. employed loss engineering to design a hook-shaped nanoantenna. Similarly, through TCMT calculations, Ren et al. discovered that nanoantennas with a high ratio of radiation to absorption losses exhibit super-sensitive reflection signals (Figure 4c) [52]. Therefore, Ren et al. adjusted the size of the hooked nanoantenna to configure its radiation-to-absorption loss ratio to be greater than one to obtain the best sensitivity.

The aforementioned studies only discussed the influence of the ratio of radiation to absorption losses on SEIRA sensitivity. During this process, the gaps between adjacent antennas were kept wide to avoid coupling between neighboring antennas. This indicates that further enhancement of SEIRA sensitivity is possible. One conceivable approach is to combine structural optimization with loss optimization to further improve sensing sensitivity. For example, Hwang et al. integrated the device’s loss parameters, near-field enhancement, and sensing area to achieve an unprecedented level of ultra-sensitive molecular detection platform (Figure 4d) [181]. This platform consisted of a metal-insulator–metal structure with a radiation-to-absorption loss ratio of 0.54. Additionally, the platform had a bottom cut with a thickness of 10 nm, effectively creating vertical nanogaps between the top nanoantenna and the bottom backplane. By utilizing the vertical nanogaps in the platform, the authors simultaneously achieved high absorption intensity, strong near-field enhancement, and a large effective sensing area. In the synergistic effect of these favorable conditions, the sensing platform obtained a record-breaking high reflectance difference in the SEIRA signal.

Similarly, Zhou et al. simultaneously considered the device’s loss parameters, near-field enhancement, and spatial overlap in a monolayer plasmonic nanoantenna to develop a CO_2_ infrared sensor with a detection limit as low as sub-ppm [157]. Within the theoretical framework of TCMT, the authors designed a monolayer plasmonic nanoantenna with multiple hotspots. The platform had a radiation-to-absorption loss ratio of 1.35, at which the SEIRA sensitivity of the reflection spectrum reached its maximum value. Furthermore, by reducing the antenna gap to 30 nm, high near-field enhancement was achieved. The spatial overlap between gas molecules and hotspots was enhanced by using a MOF/polymer molecular enrichment membrane. Through the combined effects of various optimization methods, a competitive IR CO_2_ sensor was achieved, including a 1 ppm detection limit, a high sensitivity of 0.18%/ppm, and a nanoscale optical interaction length.

Suppressing noise signals can also enhance sensitivity. To address this, Zhou et al. proposed a dual-phase enhancement strategy based on loss engineering (Figure 4e) [182]. This strategy simultaneously utilized the EIT effect and the EIA effect in the absorber. As the signals under these two effects have opposite directions, the noise in the detection system is effectively suppressed when the two counter-directional signals are combined. Additionally, the dual-phase strategy is based on system-level optimization using loss engineering, which improves the efficiency of infrared energy transfer to the molecules without requiring any additional manufacturing complexity. Therefore, it overcomes the trade-off between performance and manufacturing costs. This work presents a novel differential enhancement sensing approach and provides new insights for various sensing applications based on metamaterials.

Loss engineering is also applicable to BIC. For example, Aigner et al. designed plasmonic nanofin metasurfaces by breaking the in-plane symmetry (Figure 4f) [183]. This metasurface supports symmetry-protected BIC up to the fourth order in the continuum. By finely tuning the angles of the nanofins, precise control over the ratio of radiative losses to intrinsic losses was achieved. This enables BICs to access UC, CC, and OC states. By utilizing different coupling modes for sensing, Aigner et al. demonstrated the strong dependence of the sensing performance of BICs on the coupling mechanism. This work highlights the crucial importance of tailored coupling conditions for high-performance molecular sensing using metasurfaces with high-Q factors.

Unlike traditional structural optimization, loss engineering does not directly design the device structure but predicts the optimal parameter configuration for maximizing the SEIRA signal theoretically. This includes the radiation-to-absorption loss ratio and spectral detuning of the system. Here, Li et al. comprehensively revealed the general rules of plasmon-molecule coupling based on TCMT, which have universal applicability [54]. These rules provide important guidance for the subsequent customization of metamaterial structures. Based on these rules, nanoantenna structures can be adjusted to achieve optimal parameter configurations. The combination of loss engineering with structural optimization holds the promise of unprecedented sensitivity. Additionally, integrating loss engineering as a constraint and combining it with ML for automatic iteration of device structures can optimize the device design for optimal performance in a shorter time and with lower manual effort. In summary, as an emerging approach for optimizing SEIRA performance, the full potential of loss engineering has yet to be fully explored.

## 4. SEIRA Bandwidth

In SEIRA devices, bandwidth is a crucial performance parameter that directly reflects the detection range [184,185]. A wider spectrum allows for the collection of more fingerprint information from a broader range of molecules. Currently, various methods have been developed, including fractal geometry [186,187,188], asymmetric structures [40,189], self-similar structures [47,190], and supercells [52], to achieve multiband or broadband resonance. For instance, Gottheim et al. proposed the use of Cayley tree fractal structures to generate multi-frequency electromagnetic responses [191]. The number of resonance peaks can be controlled by the geometric iteration of the Cayley tree. Garoli et al. prepared a fractal-like plasmonic metamaterial using nanoporous gold. Among them, the plasma frequency depends linearly on the fractal dimension, which can be controlled by changing the size of the pores and ligaments of nanoporous gold [192,193]. Aslan et al. introduced a novel multiscale resonant structure based on the inverse Cesaro space-filling fractal curve [188]. This structure exhibits multiple controllable plasmonic resonances in the near-infrared to mid-infrared spectral range. The multispectral behavior of fractal geometry provides opportunities for multi-fingerprint detection. However, the resonance patterns of fractal geometry are complex, and it is challenging to achieve independent tuning of a single resonance peak. Additionally, as the fractal order increases, the coupling between different structures becomes more pronounced. The interference introduced by the mutual coupling of structures leads to energy shifts or degradation of intensity in certain resonance behaviors, limiting their applications.

**Figure 5:**
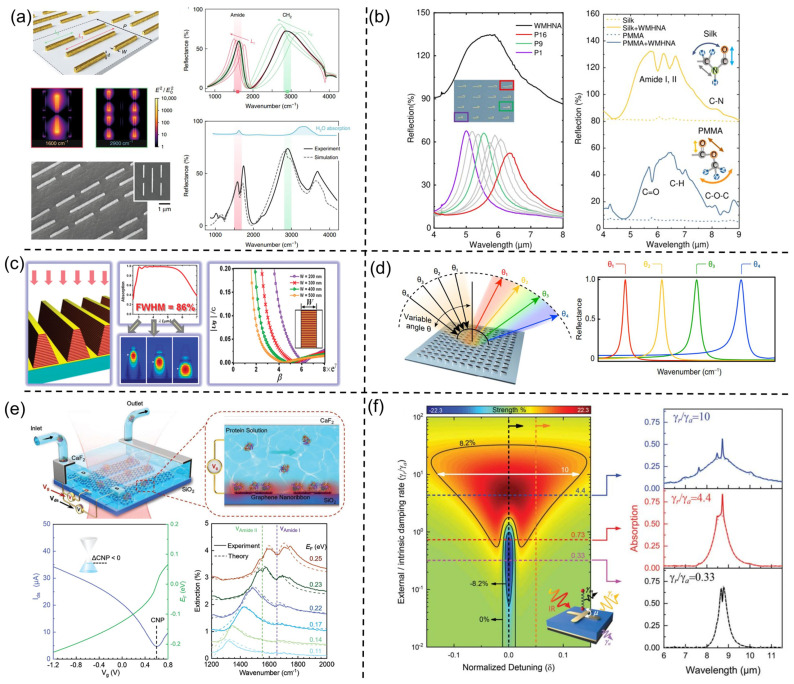
Broadand SEIRA devices. (**a**) Multi-resonant mid-IR metasurface. Reprinted from ref. [47] with permission, Copyright@2018 Optical Springer Nature; (**b**) The reflect spectra of wavelength-multiplexed hook nanoantennas (WHHNA) to form the supercell and the sensing characterization of WMHNA by two types of thin films of PMMA and silk. Reprinted from ref. [52] with permission, Copyright@2022 Springer Nature; (**c**) Ultrabroadband thin-film infrared absorber made of sawtoothed anisotropic metamateria. Reprinted from ref. [196] with permission, Copyright@2012 American Chemical Society; (**d**) Angle-multiplexed broadband fingerprint retrieval. Reprinted from ref. [51] with permission, Copyright@2019 AAAS; (**e**) Ultrasensitive mid-infrared biosensing in aqueous solutions with graphene plasmons. Reprinted from ref. [202] with permission, Copyright@2021 Wiley-VCH GmbH; (**f**) Ultrasensitive molecular fingerprint retrieval using strongly detuned overcoupled plasmonic nanoantennas. Reprinted from ref. [54] with permission, Copyright@2023 Wiley-VCH GmbH.

To pursue stable and independently tunable multiband spectra, Rodrigo et al. proposed a novel method for generating efficient and multifunctional multiscale self-similar arrays of multiresonant structures (Figure 5a) [47,190]. This method utilizes the combination of independent subarrays composed of different plasmonic nanorods to achieve multiple resonances, with each subarray providing a distinct resonant frequency. The independent resonance modes enable each spectral amplitude to reach above 70%. Furthermore, by modifying individual geometric antenna parameters, each individual resonance wavelength can be independently tuned within a 50% spectral range, offering flexible control over collective spectral responses. As a demonstration, Rodrigo et al. designed devices with up to four independently distinct resonances, covering an unprecedentedly wide spectral range from mid-infrared to near-infrared wavelengths (10–1.5 μm). Due to the broad-spectrum coverage, Rodrigo et al. further showcased the potential of the device in polymer molecule detection and dynamic monitoring of biomolecules. In addition, Li et al. were inspired by self-similar structures and designed polarization-insensitive multiresonant nanoantennas [194]. This method integrates multiple cross-structures within a unit cell using dislocation, with each structure allocated a specific resonance mode, thereby achieving 2–4 independently tunable plasmonic resonance peaks. Based on multiple resonance peaks, Li et al. achieved polarization-free multispectral detection, quantitative monitoring, and in situ reaction monitoring of polymers.

However, placing multiple antenna structures supporting different frequencies within smaller unit cells presents significant challenges. As the antenna density within the unit cell increases, the coupling between adjacent antennas also increases. Additionally, due to the gaps between two resonance peaks, the individual resonances of plasmonic nanoantennas supporting different subharmonic modes cannot cover the entire spectrum of the infrared fingerprint wavelength range from 5.5 μm to 10 μm. To address this, Ren et al. proposed a supercell design (Figure 5b) [52]. The supercell consists of 16 sub-cells, with each sub-cell containing a hook-shaped nanoantenna. These 16 hook-shaped nanoantennas have different structural sizes, supporting distinct resonance frequencies. As each hook-shaped antenna has its own independent sub-cell, it reduces the coupling between adjacent antennas. Through careful design, the combination of independent resonance peaks enables a continuous, wide spectral response from 5 μm to 7.8 μm. This broadband spectral response is crucial not only for sensors but also for improving the conversion efficiency of light absorbers or photodetectors.

The aforementioned methods achieve multi-band and broadband operation by inserting multiple resonators within the same plane. However, these methods require numerous resonators, resulting in a larger footprint and higher resonator density. Another approach to achieving broadband absorption is through the utilization of interference theory [195]. As shown in Figure 5c, researchers employed weakly coupled resonant slow-light waveguide modes within a metamaterial slab to achieve broadband absorption [196]. This anisotropic metamaterial sawtooth not only operates independently as a set of ultra-short vertical waveguides supporting different slow-light modes, capturing incident light of different wavelengths at different positions of the tooth width, but also facilitates anti-reflection of the incident light due to the gradual change in the effective index. From bottom to top, the widths of the sawtooth antennas gradually increase. As a result, light of different wavelengths is concentrated at different regions of the sawtooth absorber, thus achieving ultra-wideband absorption.

Complex nanophotonic structures have the potential to provide carefully tailored optical responses for a range of applications. However, with the emergence of such flexibility, a vast design space that is challenging to effectively harness comes along. While incorporating many different subunit elements in photon structures is desirable, the design cost exponentially increases as the dimensionality of the design space grows [197]. The introduction of machine learning has effectively overcome the aforementioned challenges. For instance, Jiang et al. utilized a genetic algorithm to design an efficient dual-band metamaterial absorber [198]. The absorber exhibited two nearly perfect narrow absorption bands centered at mid-infrared wavelengths of 3.3 μm and 3.9 μm. Subsequently, Bossard et al. employed a genetic algorithm to design a broadband, polarization-insensitive metamaterial absorber [199]. The absorber covered a wavelength range from 1.17 μm to 4.81 μm, with a measured average absorption exceeding 98% and maintaining high-efficiency absorption over a wide field of view of ± 81°. Yeung et al. demonstrated a cascaded residual network approach to efficiently generate multiplexed supercells through inverse design [200]. By utilizing a training dataset with thousands of full-wave electromagnetic simulations in a design space with over three trillion possibilities, the deep learning model accurately generated multiband/broadband structural designs.

Discrete spectral stitching to create a continuous spectrum is another strategy for achieving broadband performance. For example, Tittl et al. designed a pixelated, all-dielectric metasurface [85]. Each meta-pixel featured a high-Q narrowband resonance supported by a BIC driven by a subwavelength cavity mode. By varying the scaling of the unit cells and antennas within each meta-pixel, the resonance frequency could be linearly tuned in the mid-infrared range. This configuration allowed assigning specific pixels to each resonance position, establishing a one-to-one mapping between spectral and spatial information. This one-to-one mapping combined discrete frequencies into a continuous spectrum, enabling target molecule identification and spectral multiplexing. Importantly, this method converted spectral information into a spatial absorption pattern resembling a barcode, providing a novel solution for miniaturized infrared spectrometers. However, this one-to-one mapping resulted in larger-sized devices. To address this issue, Leitis et al. introduced the concept of angle multiplexing based on the all-dielectric metasurface (Figure 5d) [51]. This method transformed the spatial mapping relationship into a mapping between the spectrum and the incident angle of the infrared light. By controlling the incident angle of light, strong enhancement of the electromagnetic near-field and external tuning of the resonance frequency were achieved. When illuminated with incident angles ranging from 13° to 60°, a single-pixel metasurface could provide more than 200 resonances. These resonances were discretely distributed between 1100 cm^−1^ and 1800 cm^−1^, resulting in broad spectral coverage.

Metasurfaces provide a means to manipulate resonance responses. While passive metasurfaces can achieve wavelength shifts in resonances, precise control of enhancement effects within specific spectral ranges presents significant challenges due to the requirement for fine-tuning subwavelength device dimensions. However, active tuning mechanisms offer a solution, enabling fine adjustments without manufacturing limitations. Among tuning mechanisms, electrical tuning stands out for its fast response and ease of operation [201]. In recent developments, active tuning of graphene plasmon resonances has been applied to mid-infrared optical devices. In 2015, Rodrigo et al. reported a tunable graphene biosensor based on graphene plasmons [126]. By applying bias to the system, the doping level of graphene could be altered, resulting in a shift in the resonance wavelength. The intriguing electrical properties of graphene, particularly its tunable Fermi level, make it an excellent candidate for electrically tunable applications. Subsequently, Wu et al. successfully developed a tunable graphene-plasmon-enhanced aqueous spectroscopic sensing system (Figure 5e) [202]. An important aspect of their study was the dynamic modulation of plasmon resonance frequency. By tuning the Fermi level of graphene from approximately 0.11 eV to 0.25 eV, the plasmon resonance frequency could be dynamically adjusted between approximately 1300 cm^−1^ and 1700 cm^−1^. This tunability enables precise control of the system’s spectral response, thus achieving broadband spectra.

Another approach to achieving tunability is by integrating grating materials (such as conductive polymers) that exhibit dynamically changing optical properties under an external electric field. Karst et al. demonstrated a study where they utilized optically metalized polymers to showcase electrically switchable nanoantennas [203]. The polymer exhibited an electrochemically driven metal-to-insulator transition within the near-infrared spectral range, which was induced by changes in carrier density. This transition allowed for modulation of the polymer’s optical properties, enabling control over the resonance characteristics of the nanoantennas. By controlling the applied electric field, the carrier density could be altered, resulting in tunable and switchable behavior of the nanoantennas within the desired spectral range. This dynamic control over carrier density and the optical properties of the polymer allows for the realization of highly tunable and switchable systems, enabling effective modulation of the behavior of the nanoantennas.

Although the combination of multiple resonances and modulation can increase the bandwidth of plasma resonance, the problem of low light–matter interaction in the broadband range hinders its widespread application. Recently, Li et al. comprehensively revealed the principles of plasma-molecule interaction through TCMT and found that absorbers in the OC mode can achieve broadband spectral enhancement (Figure 5f) [54]. Despite exhibiting a low Q-factor and low resonance intensity, these absorbers demonstrate excellent sensing performance. These outstanding sensing capabilities include robust sensitivity, broadband spectral enhancement, and immunity to asymmetric Fano resonances. These features enable a single device to enhance and capture complete and complex fingerprint vibrations in the mid-infrared range, thereby achieving spectral multiplexing and molecular fingerprint retrieval. Based on the massive spectral data collected by the OC absorber, Li et al. introduced ML to achieve accurate identification of 13 analytes. This work provides new insights into broadband spectral enhancement and multi-analyte sensing.

## 5. Plasmonic Nanostructure-Based Modulating and Sensing Applications

After the previous introduction of SEIRA sensitivity and bandwidth, we will further discuss the potential applications of plasmonic nanostructures in this section. Apart from the applications in the mid-infrared range, we will also review the applications in other wavelengths like visible light, near-infrared, and the terahertz band. We hope this can provide the readers with a more comprehensive scope of plasmonic nanostructures while comparing the scenarios of potential applications in all wavelength bands.

### 5.1. Mechanical Modulator

Microelectromechanical System (MEMS) devices leverage the mechanical deformation of movable structures, providing tunable designs for many applications [204,205,206,207,208,209,210,211,212,213]. In optical frequencies, there are two types of MEMS devices. The first one is an optically non-resonant MEMS device. These devices leverage the movement of bulk MEMS structures to manipulate light through in-plane and out-of-plane designs [214,215,216]. One example of such a device is the MEMS mirror, where the light path can be manipulated by the rotational reflector controlled by deformable beams [214]. Although these kinds of MEMS devices are easy to design with a large tuning range, they only enable the manipulation of the wavefront of electromagnetic waves, while the modulation of light cannot be fulfilled. Fortunately, another type of MEMS device, optically resonant devices, can fulfill this function. With more interesting findings having been figured out in recent years, these resonant MEMS plasmonic devices have attracted more attention [209,213,217,218]. These structures themselves work as resonators, and resonant peaks can be observed on the spectra. Therefore, by tuning these deformable structures, the resonant mode can be changed. Regarding the mechanisms, the modulation can be implemented based on the resonance amplitude, frequency, and polarization of the electromagnetic waves. In this section, we mainly introduce several metadevices with representative resonant structures and modulation methods.

**Figure 6:**
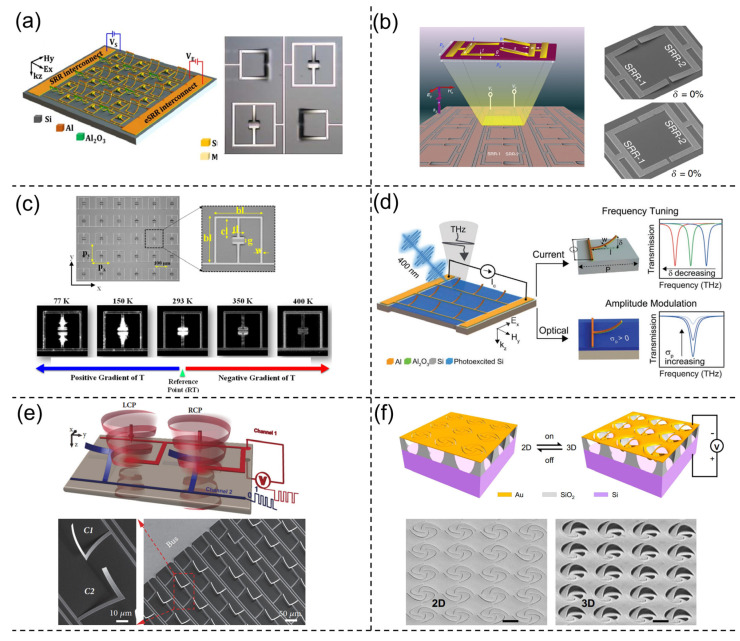
Plasmonic Microelectromechanical System (MEMS) tuning devices. (**a**) Electrostatic tunable cantilever for THz wave modulation. Reprinted from ref. [46] with permission, Copyright@2015 Optical Society of America; (**b**) Electrostatic tunable split-ring resonator (SRR) for THz logic gate. Reprinted from ref. [227] with permission, Copyright@2018 Springer Nature; (**c**) Thermally tunable cantilever for THz wave modulation. Reprinted from ref. [228] with permission, Copyright@2017 AIP Publishing; (**d**) Hybrid electrostatic-optical tunable cantilever for frequency and amplitude modulation of THz electromagnetic wave. Reprinted from ref. [234] with permission, Copyright@2020 Wiley-VCH; (**e**) Electrostatic tunable L-shaped cantilever for circular polarized light modulation. Reprinted from ref. [235] with permission, Copyright@2019 AAAS; (**f**) Electrostatic tunable kirigami for circular polarized light modulation. Reprinted from ref. [236] with permission, Copyright@2021 Springer Nature, Scale bars: 1 μm.

In 2015, Pitchappa et al. introduced a reconfigurable metamaterial for independent THz modulation composed of a multi-resonator system [46], as shown in Figure 6a. The unit cell of the proposed metadevice consists of four resonators, two of which are cantilevers that control the electrical resonance and the other two are Split-Ring Resonators (SRR) that control the magnetic resonance. Hence, by applying two voltages to induce electrostatic forces, the resonant mode of the cantilever and SRR can be controlled independently, ranging from 0.2 THz to 0.7 THz. This kind of MEMS-based metadevice has been widely proposed for different tuning mechanisms and explored for various functionalities [219,220,221,222,223,224,225,226]. Manjappa et al. further leverage such technology and propose logic functions for free-space communications at THz frequencies [227], as shown in Figure 6b. The resonant system is simplified into two SRRs, which are independently controlled by applying different voltages. Therefore, by controlling the released heights of the two SRRs, the changed resonant states can be utilized for realizing logic functions like XOR and XNOR. The operation of the logic features at THz frequencies can be significant in cryptographic wireless communication networks for 6G applications. Apart from electrostatically actuated MEMS devices, another method is to leverage thermally induced deformations. Pitchappa et al. conducted such a demonstration by applying a thermal gradient from 77 K to 400 K to cantilever-based MEMS metadevices [228], as shown in Figure 6c. The cantilever undergoes significant deformation when the temperature changes and the near-field coupling between the cantilevers is tuned, resulting in a frequency shift from 0.32 THz to 0.42 THz. However, one of the disadvantages of thermally driven devices is their low response time, which makes it difficult to serve high-speed applications, especially when compared with ultrafast tuning materials such as GST and photo-sensitive materials [229,230,231,232,233]. Regarding the request for fast response time, Pitchappa et al. developed a hybrid tuning mechanism recently [234], as shown in Figure 6d. Different from previous work, they proposed both electrically and optically tuned methods to provide frequency and amplitude modulation separately. This hybrid tuning method increases the tunability of metadevices by enabling amplitude tuning at an arbitrary frequency and providing more flexibility in MEMS structural designs.

Apart from amplitude and frequency, another important characteristic of light is polarization. Pitchappa and his co-authors demonstrated the manipulation of linear-polarized light using similar building blocks consisting of four cantilever beams [222], illustrating the basic design framework for controlling linear-polarized light with independent tuning methods. However, the manipulation of circular-polarized light can be more challenging, as the structure needs to present an unequal response to different circularly polarized states, known as optical chirality [237,238]. To solve this problem, Cong et al. proposed “L”-shaped cantilevers for such functionality [235], as shown in Figure 6e. The “L”-shaped structures are oriented with a mirror-plane symmetry, where they can be controlled independently by applying voltages. After being deformed by electrostatic forces, the cantilevers will have opposite bending angles, which will induce different external chirality due to the out-of-plane symmetry-breaking modes. Such structures enable circular polarized light manipulation in THz wavelengths, which has the potential for polarization-multiplexed communication applications. These methods all enable the modulation of THz beams, which is limited by the scaling factors of the cantilevers and SRRs. Bring the technology to shorter wavelengths; such structures require larger power consumption and smaller critical dimensions, which can be challenging for the lithographically patterned meta-structures. Therefore, nanostructures with higher efficiency are desired for higher optical frequencies. Chen et al. proposed a nano-kirigami array, bringing electrically tunable structures composed of spiral and pinwheel patterns [236], as shown in Figure 6f. Although the tunability of such a kirigami structure is low with high electrical power consumption, the device can realize the control of Circular Dichroism (CD) at Near-Infrared (NIR) wavelengths by deforming the structures between 2D and 3D states. Moreover, the fabrication steps are also easy to operate, with the possibility of mass production. There have also been works focusing on extending the functionalities of plasmonic nano-kirigami structures [239]. However, the Ohm loss of the plasmonic materials hinders potential commercial applications. One possible solution is to leverage dielectric low-loss structures to substitute the plasmonic materials, which require further development for characterization [240,241].

### 5.2. Biomedical and Environmental Sensors

Plasmonic structures leverage the near-field enhancement to improve the spectroscopic response of the molecules. After introducing general principles like sensing theory and sensor structures, we will zoom in to further explore specific applications with certain targeted sensing molecules. In this section, we mainly focus on molecules related to biomedical and environmental applications.

**Figure 7:**
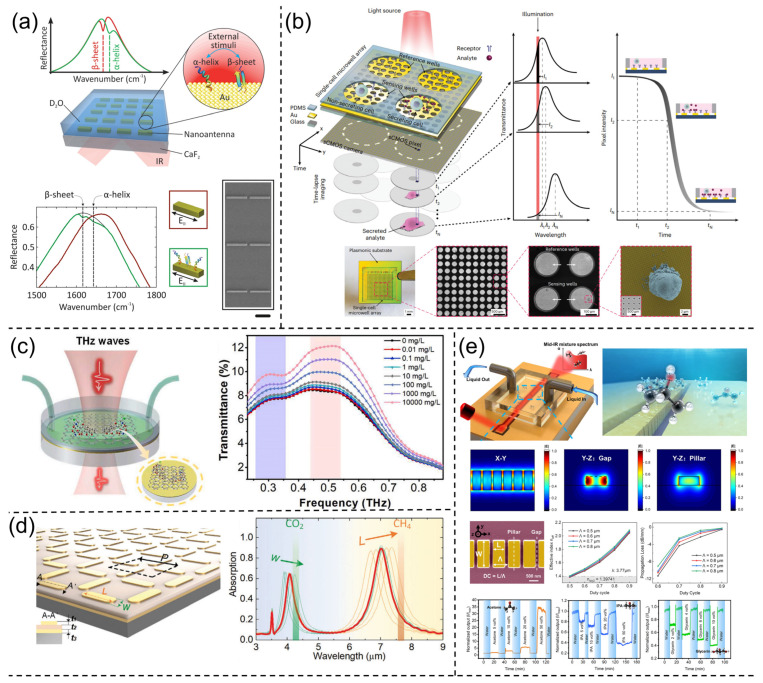
Plasmonic devices for biomedical and environmental monitoring applications. (**a**) Mid-infrared nanoantenna for protein secondary structure sensing. Reprinted from ref. [250] with permission, Copyright@2019 American Chemical Society, Scale bars: 1 μm; (**b**) Microwell array sensing of antibodies, Reprinted from ref. [58] with permission, Copyright@2023 Springer Nature; (**c**) Asymmetric SRR THz sensor for DNA sensing. Reprinted from ref. [258] with permission, Copyright@2021 Elsevier Publishing; (**d**) Mid-infrared plasmonic perfect absorber for greenhouse gas sensing. Reprinted from ref. [156] with permission, Copyright@2020 Wiley-VCH; (**e**) Subwavelength grating nanostructures for aqueous mixture sensing. Reprinted from ref. [167] with permission, Copyright@2023 American Chemical Society.

The detection of biomedical molecules plays an important role in diagnosis, healthcare monitoring, and therapy development applications [242,243,244]. These biomolecules are also known as biomarkers, and the concentration level of these molecules can indicate significant information about the patient. One of them is the secondary structure of proteins and peptides, as they contribute to biochemical reactions in living cells [245]. The misfolding and aggregation of secondary structures can result in neurodegenerative diseases like Parkinson’s disease and Alzheimer’s disease [246]. There have been many non-optical methods for characterizing secondary structures, such as Nuclear Magnetic Resonance (NMR) [247], Atomic Force Microscopy (AFM) [248], and X-ray crystallography [249]. However, these methods usually require a large sample volume for analysis, and the monitoring process is not real-time. Hence, spectroscopic monitoring of the biomarkers is worth developing. In 2019, Semenyshyn et al. proposed in vitro monitoring of conformational changes in peptides using surface-enhanced vibrational spectroscopy [250], as shown in Figure 7a. They leverage the hotspots of the nanoantennas to enhance the vibrational signal of α-helix and β-sheet absorption peaks in the sensitive amide-I region from 1600 cm^−1^ to 1700 cm^−1^. Moreover, the secondary Principal Component Analysis (PCA) method is used, which can detect the mixture cluster of both secondary structures. Hinkov et al. also proposed a lab-on-a-chip compact sensing platform for dynamic reaction monitoring with temperature-induced conformation changes, which further enables the potential of miniaturizing the sensing technology [251]. Although they lack the selectivity of molecular fingerprints, there are also other optical methods for secondary structures, such as CD spectroscopy [252,253]. Apart from proteins and peptides, antibodies are another important biomolecule for the immune systems of living creatures. The activation efficiency and specificity of the immune response require quantitative and real-time analysis at the single-cell level [254]. Ansaryan et al. proposed a label-free optical detection method that enables spatiotemporal monitoring of single-cell secretions [58], as shown in Figure 7b. By fabricating a plasmonic single-cell microwell array, the cells can be clearly monitored on the substrate, with intensity changes displayed on the transmittance spectrum. Furthermore, spatiotemporal monitoring is also demonstrated through morphological changes using machine learning algorithms. It is also worth mentioning that such proposed methods are not only limited to antibodies but also include cytokines and extracellular vesicles, making this a promising platform for biomedical applications. Moreover, it is worth noting that single-cell-level analysis can also be implemented using SEIRA nanoprobes. Domennici et al. proposed a novel method using 20 nm gold nanoparticles conjugated with biomarkers that highlight the occurrence of biological effects, providing strategies for cell spectral imaging and drug delivery-based therapies [255]. Such methods are also compatible with SERS-based platforms [256,257]. Another important biological molecule that raises significant interest is the DNA sequence, which reveals genetic information. Zhou et al. leveraged the intramolecular vibrational modes of biological macromolecules and developed a THz DNA sensor using graphene with antisymmetric SRRs [258], as shown in Figure 7c. Such hybrid graphene-metasurfaces with tunable Fermi levels enable current density modulation as well as liquid sensing of DNA macromolecules through microfluidic channels. Moreover, the sensor is also capable of sensing solutions with varied concentrations, showing the potential of dynamic sensing in chemical reactions. This type of THz sensing platform is inspiring for the characterization of both intermolecular and intramolecular signals, as THz electromagnetic waves can be non-invasive, label-free, and real-time [189,259,260,261,262,263,264].

Environment monitoring can be another important potential application of enhanced plasmonic sensors. One typical scenario is greenhouse gases composed of CO_2_ and CH_4_, whose detection enables real-time monitoring and management in industry and meteorology [265,266]. In 2020, Zhou et al. proposed a Metal–Organic Framework (MOF)-SEIRA platform and demonstrated simultaneous sensing for both greenhouse gases [156], as shown in Figure 7d. The MOF helps increase the sensitivity of gases by leveraging their porous structure and selectivity. Moreover, the metamaterials are designed with multiple resonances, where the resonance peak matches the vibrational modes of CO_2_ and CH_4_, respectively. Therefore, the authors realize dynamic sensing of the gases with a ppm level detection limit. However, one severe limitation of gas sensing through MIR spectroscopy is the interference by water vapor, and hence, there have been efforts to minimize the influences [267,268,269]. Another scenario for aqueous environment detection, however, diminishes this influence. Recently, Zhou et al. reported an on-chip MIR sensing platform for liquid mixtures [167], as shown in Figure 7e. The authors leverage Subwavelength Grating (SWG) structures as metamaterial waveguide sensors to create more evanescent waves where the molecules can experience larger field enhancement in the nanogap region. Based on the enhanced sensor, a microfluidic chamber is integrated for liquid sensing demonstration. In addition, machine learning algorithms are also utilized for processing the abundant sensing data. The whole sensing system shows a low detection limit, high sensitivity, and high accuracy for the classification of ternary mixtures with mixed volume ratios, which is promising for monitoring components of liquid mixtures in complex environments. As a further development, mixture sensing is believed to have more potential applications in this field, such as the detection of biomarkers in blood, urine, and saliva [270,271,272]. Moreover, on-chip compact plasmonic devices are also significant for industrial or commercial needs, including compact integration of plasmonic devices and sensing chambers [52,53], and a compatible user interface for signal post-processing [273,274].

### 5.3. Chiral Sensor

Chirality describes objects that cannot be superimposed on their mirror image. In stereochemistry, molecules can present chirality when there is a chiral center, which is a spatial asymmetry [237,275,276]. In chemical reactions, if the molecules contain chiral isomers, there may also be enantiomers in the product. Moreover, the existence of such an enantiomer may affect its chemical and biological properties, especially in drug production [277,278,279]. Therefore, it is important to develop methods to detect chiral molecules. However, due to identical chemical bonds, it is difficult to distinguish them through neither the real part nor the imaginary part of the refractive index. Fortunately, based on the interaction with the circularly polarized light, Circular Dichroism (CD) spectroscopy and Optical Rotatory Dispersion (ORD) spectroscopy stand out in detecting the chiral light–matter interaction between Left-handed Circularly Polarized light (LCP) and Right-handed Circularly Polarized light (RCP). Moreover, the plasmonic nanostructure can be further utilized to enhance the intrinsically weak chiral signals of chiral molecules by leveraging an enhancement of the optical chiral field, which is also known as the superchiral field [252,275,280]. The optical chiral field can be determined as [275]:

(3) $$C=\frac{\varepsilon_{0}}{2}E\cdot \nabla \times E+\frac{1}{2\mu_{0}}B\cdot \nabla \times B$$

where *ε*_0_ and *μ*_0_ are the permittivity and permeability of the vacuum, respectively. ***E*** and ***B*** represent the local electric and magnetic fields. For circularly polarized light propagating in free space, the chiral field *C* can achieve a maximum value of 1 and a minimum value of −1, which represent the spin states of LCP and RCP lights, respectively. Combined with plasmonic structures, the near-field enhancement can enable the boost of the localized chiral field to break the limitation of ±1 [280], which enhances the signal of chiral molecules.

**Figure 8:**
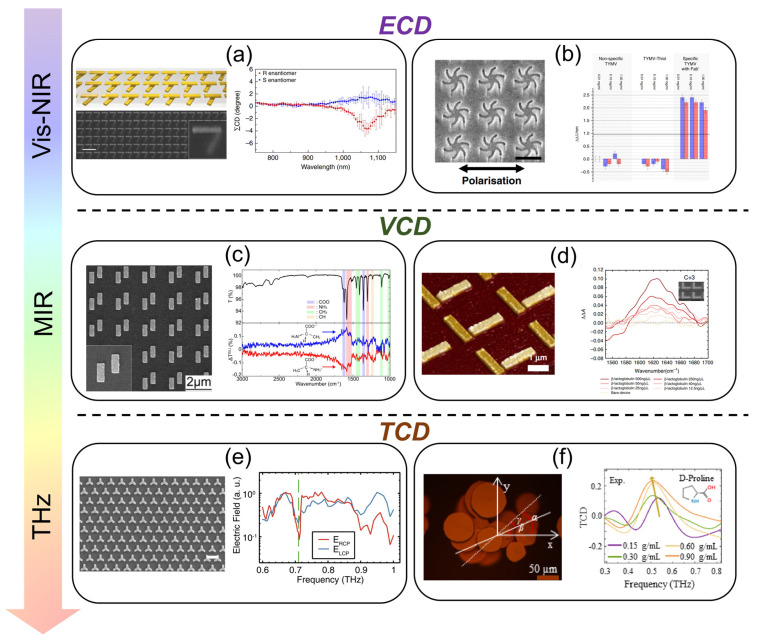
Plasmonic chiral nanostructures for chirality sensing applications. (**a**) Stacked chiral metamaterials for enantiomer sensing applications using CD spectroscopy. Reprinted from ref. [283] with permission, Copyright@2017 Spring Nature, scale bar is 500 nm; (**b**) Shuriken-typed chiral metasurface for virus detection application using CD spectroscopy. Reprinted from ref. [286] with permission, Copyright@2020 Spring Nature, Scale bar is 550 nm; (**c**) Double nanorod chiral metasurface for enantiomer sensing applications using VCD spectroscopy. Reprinted from ref. [288] with permission, Copyright@2020 AIP Publishing; (**d**) Perpendicularly-positioned chiral metamaterials with different thicknesses for protein secondary structure sensing applications using VCD spectroscopy. Reprinted from ref. [292] with permission, Copyright@2023 Spring Nature; (**e**) Nonlinear emission of chiral light and detection of enantiomers using TCD spectroscopy, Reprinted from ref. [299] with permission, Copyright@2021 Spring Nature, Scale bar is 550 nm; (**f**) Multilayered plasmonic resonators for enantiomer sensing applications using TCD spectroscopy. Reprinted from ref. [300] with permission, Copyright@2022 Optical Society of America.

The commonly used CD spectroscopy locates at shorter wavelengths due to stronger molecular CD signals, ranging from UV to NIR. The chiral metamaterials can be classified as planar ones and out-of-plane ones, where the out-of-plane ones can present a larger optical chirality due to the extrinsic chirality [281]. However, for chiral molecule sensing applications, near-field enhancement can be more important, as achiral dielectric resonators can also present enhanced molecular signals [282]. In 2017, Prof. Alu’s team proposed a stacked chiral metamaterial, where the structure is composed of multiple layers [283], as shown in Figure 8a. Such chiral metamaterials create a chiral signal by leveraging the angles between two layers of nanoantennas. When the chiral analyte flows onto the top surface of the chiral metamaterial, it will experience chiral field enhancement. Additionally, the angle between nanoantennas can be engineered to enhance both left-handed molecules and right-handed molecules when coated onto different devices. This type of stacked metamaterial has been further explored with more interesting properties for more than sensing applications [284,285]. Another type of planar metamaterial has been reported earlier by Prof. Kadodwala’s group in 2010, where the gammadion structure is first proposed to generate an enhanced superchiral field, which has the potential for secondary structure sensing applications [252]. The work proposed by the same group is shown in Figure 8b [286]. The shuriken-shaped metasurface not only leverages the superchiral field enhancement but is also accompanied by immobilization strategies to improve the specific binding between substrate and oriented molecules. These results suggest the potential detection of more specific molecules when combined with both superchiral fields and substrate engineering. Expanding the wavelengths to longer ranges, the electrical CD is equipped with additional information known as Vibrational Circular Dichroism (VCD) [287]. Different from ECD spectroscopy, VCD also enables the observation of the vibrational transition of chiral molecules and can provide more information. However, the signal is not as strong as in ECD spectroscopy. Therefore, it is worth developing enhanced VCD spectroscopy using plasmonic metamaterials. Knipper et al. and Iida et al. proposed slit-enhanced and nanorod pair structures to provide enhanced VCD signals, respectively [288,289]. Their works revealed the possibility of using chiral plasmonic structures to selectively enhance the molecular VCD signals, as shown in Figure 8c. It can be noticed that the VCD signals are associated with the vibrational peaks of the chiral molecules, providing extra information to distinguish both the identity and the chirality of the enantiomers. With such a property, it is possible to help distinguish chiral mixtures using the enhanced VCD spectrum, which is highly potential and desired in biomolecule sensing applications [290,291]. Recently, Xu et al. proposed a mid-infrared chiral metamaterial device with both out-of-plane and in-plane symmetry-breaking designs, which can enhance the weak VCD signal of protein secondary structures [292], as shown in Figure 8d. Moreover, this work demonstrated for the first time the possibility of using surface-enhanced VCD to detect chiral mixtures, which expanded the potential of VCD spectroscopy. Additionally, combined with Raman spectroscopy, the Raman Optical Activity (ROA) can also be beneficial from the vibrational transitions and used for ultrasensitive sensing applications [293,294,295]. The extended wavelength to THz will further introduce Terahertz Circular Dichroism (TCD) for wider sensing applications [296,297,298]. Recently, it has been demonstrated that the THz intermolecular vibrations can be coupled with chiral phonons in microcrystals for multiple molecule detection [296], attracting much attention to this research area. One interesting phenomenon is to leverage nonlinear metasurfaces to generate THz waves with different circular polarizations, as proposed by McDonnell et al., as shown in Figure 8e [299]. Leveraging the nonlinear effect of the C3 metasurface, the THz wave is more strongly absorbed by the chiral molecules after generation from the P–B phase metasurface, enabling a multi-function emitter as well as a sensor. Zhang et al. also proposed a new type of TCD sensor composed of four-layered gold-disk metamaterials and demonstrated the sensing performance with varied concentrations of D-proline analytes, as shown in Figure 8f [300]. The THz absorption of molecules paves the way for label-free enantioselective sensing in the THz region but requires further development as there is still a lack of a design framework and comprehensive study for the sensing capability.

## 6. System Integration

Infrared spectroscopy emerges as an influential technique capable of acquiring information regarding the chemical structure and substance type of the target without the need for destructive methods or labeling. After the previous review of plasmonic nanostructure-enhanced infrared spectroscopy in single optical components, we focus on the significant role of plasmonic nanostructure in the integration and miniaturization of optical systems. Typically, a complete optical system comprises four essential components: a light source, sensors, filters, and photodetectors [301,302,303,304]. Nonetheless, traditional optical devices tend to involve numerous bulky components, resulting in large device volumes and posing challenges for on-chip integration [305]. To overcome this limitation, the miniaturization of infrared spectroscopy has become increasingly desirable, holding the potential to revolutionize the field and open up new applications in areas like the Internet of Things (IoT) and sensor networks. Achieving miniaturization in infrared spectroscopy entails reducing the size of each individual component while maintaining satisfactory overall sensitivity. However, striking a balance between size and performance often proves to be a challenging task.

**Figure 9:**
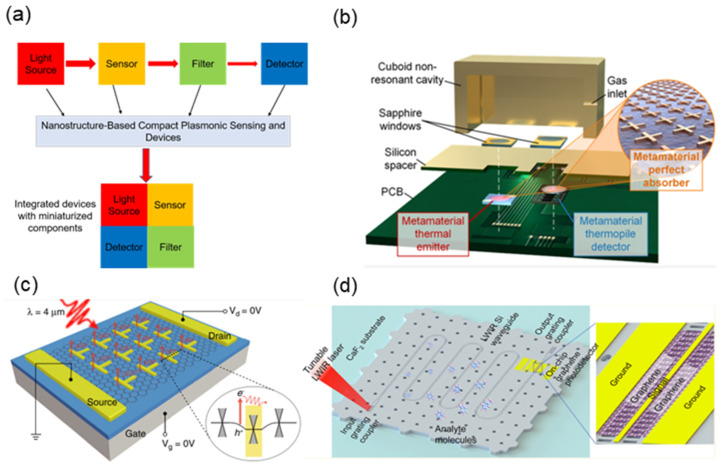
System integration. (**a**) Possible schematic for ultracompact integrated spectrometry; (**b**) Recent advances that leverage the common platform of metamaterials to shrink the size of infrared spectrometry. Reprinted from ref. [57] with permission, Copyright@2020 American Chemical Society; (**c**) Metasurface-mediated graphene polarization detectors. Reprinted from ref. [308] with permission, Copyright@2020 Springer Nature; (**d**). Heterogeneously Integrated Graphene/Silicon/Halide Waveguide Photodetectors toward Chip-Scale Zero-Bias Long-Wave Infrared Spectroscopic Sensing. Reprinted from ref. [314] with permission, Copyright@2021 American Chemical Society.

Metamaterials present unique opportunities for miniaturized infrared spectroscopy [33,306,307]. As depicted in Figure 9a, by leveraging the advantages of metamaterial-based nanostructured compact plasmonic devices, the miniaturization of infrared spectroscopy can be realized, paving the way for integrated and versatile devices in this field. As illustrated in Figure 9b, Lochbaum et al. conducted a study where they demonstrated an all-dielectric optical gas sensor that integrated optical filters, an emitter, and a detector [57]. This innovative design resulted in a significant reduction in absorption volume, achieving a 30-fold reduction compared to conventional gas sensors. Remarkably, the all-dielectric sensor exhibited a CO_2_ sensitivity of 22.4 ± 0.5 ppm·Hz^−1/2^. This impressive performance highlights the potential of all-dielectric systems for highly sensitive and compact gas-sensing applications. For the photodetectors integration, Wei et al. proposed filterless Metasurface-Mediated Graphene Photodetectors (MMGPDs) for polarization detection [308]. These photodetectors exhibited polarization-dependent photovoltage, resulting from the artificial anisotropy created by finely designed nanoantennas with broken asymmetry. Remarkably, the MMGPDs demonstrated high responsivity and low noise-equivalent power, reaching as low as 0.12 nW Hz^−1/2^ under zero bias. Building upon their previous work, they further advanced the MMGPDs by incorporating droplet-shaped nanoantenna components as metaatoms and extending their application to circular polarization [309]. Through precise control of the angles of these components, they achieved tunable polarization ratios from −∞ to −1 and from 1 to +∞, which is a crucial figure of merit in polarization detection. By using the T-shaped nanoantenna, they have demonstrated a high discrimination ratio between left circular polarization and right circular polarization [310].

Xie et al. further extended the operational wavelength of Nanoantenna-Mediated Graphene Photodetectors (NMGPDs) to the Long-Wave Infrared (LWIR) and chip-level LWIR NMGPDs as an integrated platform for polarimetric and spectroscopic sensing [311]. LWIR processes have enormous potential for chem/biosensing as they cover abundant absorption fingerprints of gas molecules and biomolecules, which can be used as biomarkers for healthcare monitoring and early disease diagnosis [130,312]. Conventional optical sensors require another photodetector (like NDIR) or a bulky spectrometer to collect the sensing information. Compared to these conventional optical sensors, Xie et al. integrated the gas sensing and polarization detectors together by utilizing a hybrid of graphene and nanoantenna.

Another promising candidate for on-chip integration is waveguide-based devices, where optical waveguides are tightly packed onto a single chip, enabling sensitive molecule detection in a compact form [306,313]. Ma et al. proposed LWIR waveguide-integrated photodetectors through heterogeneous integration of graphene photodetectors and Si waveguides on CaF_2_ substrates [314]. The waveguide demonstrated low loss over a broadband range from 6.3 to 7.1 μm. Leveraging waveguide integration and plasmonic enhancement, the graphene photodetector achieved a broadband responsivity of approximately 8 mA/W at these low-photon-energy LWIR wavelengths under zero-bias operation. By integrating the graphene photodetector with a Si-on-CaF_2_ folded waveguide, they successfully demonstrated on-chip absorption sensing using toluene as an example.

## 7. Conclusions

We have discussed numerous ways to achieve SEIRA spectroscopy, ranging from metal island films with the SEIRA effect to customizable metamaterials and their widespread applications today. This review primarily focuses on the materials, sensitivity, bandwidth, applications, and system integration of the SEIRA effect. Materials that achieve the SEIRA effect include metals, dielectrics, low-dimensional materials, and phonon resonance materials. Each of these materials possesses distinct characteristics. These materials enrich the extensive family of metamaterials, but each material also has its limitations. By combining two or more materials, it is possible to overcome the drawbacks of each material and generate new functionalities. These characteristics present new opportunities for controlling light propagation and infrared sensing.

Sensitivity and bandwidth are two critical figures of merit that reflect the performance of SEIRA. Improving sensitivity allows the detection of smaller quantities of molecules, enabling various applications, including medical diagnostics and environmental monitoring. Currently, the main approaches to improving SEIRA sensitivity include enhancing near-field intensity, increasing the spatial overlap between molecules and the near field, and optimizing losses. Additionally, dielectric materials capable of achieving BIC and graphene with highly confined optical fields offer new opportunities for enhancing SEIRA sensitivity. Due to the intricate nature of molecular fingerprint vibrations, enhancing SEIRA bandwidth can provide access to more vibrational information, thereby enabling molecular retrieval. To achieve multi-band/broadband resonances, various design methods have been proposed, including fractal geometry, asymmetric structures, self-similar structures, supercells, pixelated metasurfaces, and electrically modulated metasurfaces. Customizing metamaterials through loss engineering also provides new avenues for constructing broadband spectra. Moreover, the use of ML for the inverse design of plasmonic nanostructures with high sensitivity and broadband characteristics has gained significant attention in recent years.

Plasmonic nanostructures bridge the length-scale gap between infrared wavelengths (micrometer scale) and molecular analyte sizes (nanometer scale), opening up exciting sensing applications. Currently, sensing based on plasmonic nanostructures has covered various states of matter, including solids, liquids, and gases, and has found applications in diverse fields such as biomedicine, environmental monitoring, chemistry, materials, and more. This review focuses on the applications of plasmonic nanostructures in the fields of biomedicine and environmental detection. However, the widespread application of most metamaterials in sensing is limited by the reliance on bulky spectrometers or optical systems. To overcome this limitation, miniaturization of infrared spectroscopy has become highly desirable, as it holds the potential to revolutionize the field and enable new applications in areas like the IoT and sensor networks. Extensive efforts have been devoted to the development of small-scale spectrometers through system integration. It is envisioned that future optical systems will be highly miniaturized and integrated on-chip. Such chip-based experimental systems will facilitate broader applications in consumer technologies and wearable devices.

## Data Availability

Not applicable.
